# Supplementary material for: Deep learning-enabled multi-organ segmentation in whole-body mouse scans
Source: Nat Commun. 2020 Nov 6;11:5626. doi: 10.1038/s41467-020-19449-7 (PMC7648799; doi:10.1038/s41467-020-19449-7)
Supplement: Supplementary file 1 — Supplementary Information [file 41467_2020_19449_MOESM1_ESM.pdf]

Supplementary Figures

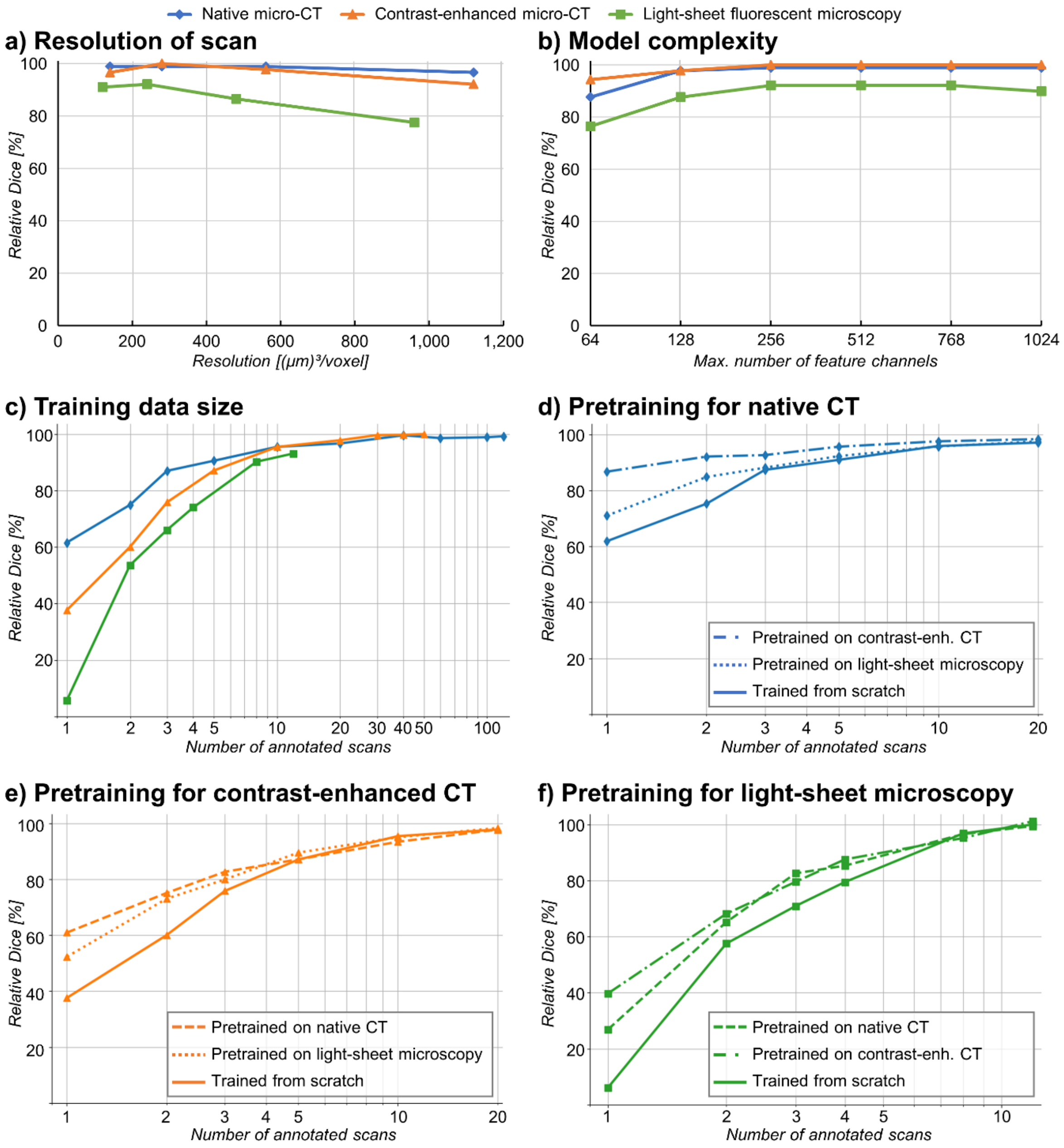

Supplementary Figure S1. Ablation study on design choices. All panels show the relative performance of AIMOS (Dice score; averaged across organs; normalised by maximum performance) as a function of a model parameter. **a)** Prediction performance is relatively stable independent of resolution of acquired imaging data (quantified as size of an isotropic voxel in µm³/vx. **b)** Model complexity, quantified as maximum number of feature channels in a U-Net-like architecture; please note that an increase in feature channels goes along with an increase of model layers (features double with each layer). **c)** Prediction performance as function of size of training data set (quantified as number of annotated whole-body mouse scans; shown in logarithmic scale) for all datasets if models are trained from scratch. **d-f)** Prediction performance if model is trained from scratch or pretrained on other datasets for native CT (**d**), contrast-enhanced CT (**e**), and light-sheet microscopy (**f**). Source data are provided as a Source Data file.

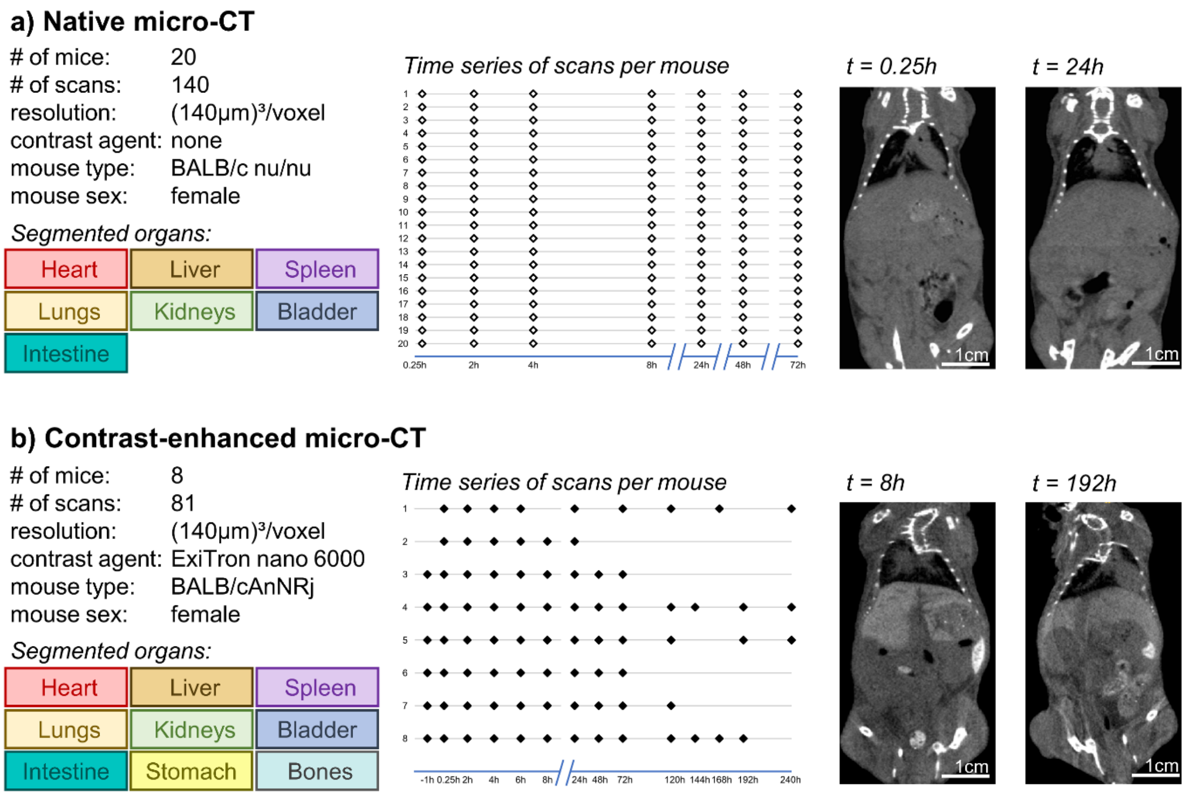

Supplementary Figure S2. Micro-CT datasets used in this study from Rosenhain et al. (2018). For each scan, the mice were newly anesthetised and positioned in a sample holder. **a)** For the native micro-CT dataset, 20 mice were scanned at 7 time points. **b)** For the contrast-enhanced micro-CT dataset, 8 mice were scanned at varying time points after injection of the contrast agent.

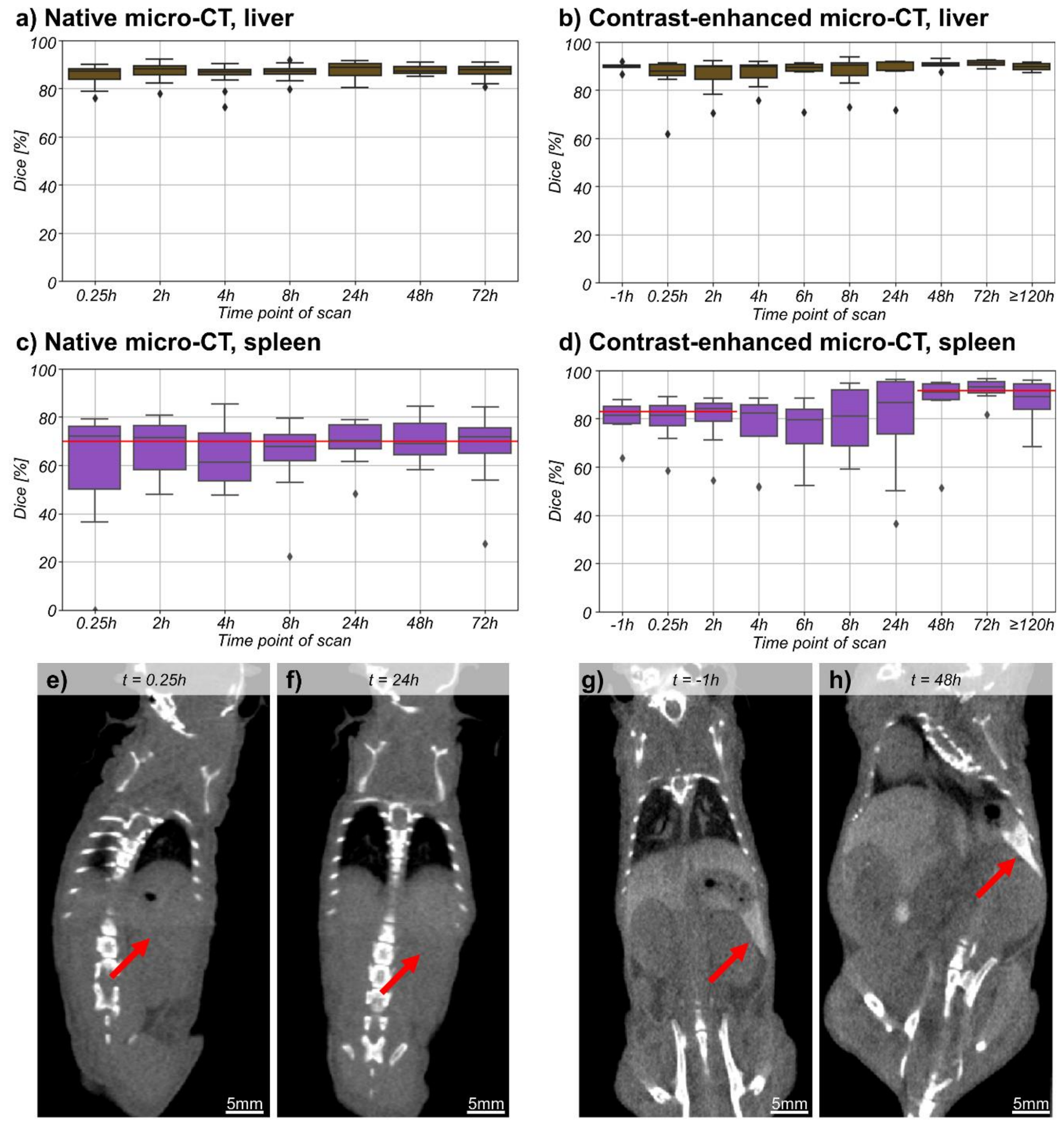

Supplementary Figure S3. Segmentation performance for liver and spleen in CT. Both organs are the main target of the contrast agent, which is reported to show peak contrast at t=4h for the liver and at t=48h for the spleen. **a-d)** Box plots of prediction performance (Dice score) per time point for both CT datasets. Each box extends from lower to upper quartile values of data, with a black line at the median; whiskers extend to outmost data point within 1.5x the interquartile range; outliers beyond whiskers are shown as diamonds. **a,b)** Each box plot represents represents n=140 independent scans from 20 biologically independent animals. For the liver, there is no difference between native CT (**a**) and contrast-enhanced CT (**b**). **c,d)** The number of independent scans and the number of biologically independent animals varies by time point and is provided in Supplementary Figure S2b). **c**) For the spleen in native CT, the median performance over all time points (70%) is indicated with a red line. **d)** For the spleen in contrast-enhanced CT, red lines show the median performance for two time periods; left: immediately after contrast agent injection (83%); right: at t = 48h or later (92%). **e-h)** Cross-sections through CT scans (brightness normalised from -500 HU to 1.000 HU). Location of spleen indicated by red arrows. **e,f)** Two exemplary scans show that the outline of the spleen is barely visible, if at all, in the native CT scan. **g,h)** Scans from the contrast-enhanced CT dataset show that the agent increases contrast over time; however, even at t = -1h (which is after injection of the contrast-agent) the spleen is already much more visible as compared to the native CT dataset. Source data are provided as a Source Data file.

Dataset	Organ	Dice score	50th percentile Hausdorff distance	95th percentile Hausdorff distance	Center of mass displacement	Dice score, w/o ensemble
**Native micro-CT**	Spleen	74%	0.2 mm	0.8 mm	0.5 mm	72%
	Heart	92%	0.1 mm	0.4 mm	0.2 mm	91%
	Lung	93%	0.1 mm	0.2 mm	0.1 mm	93%
	Liver	89%	0.3 mm	1.1 mm	0.4 mm	88%
	Kidney (left)	89%	0.1 mm	0.6 mm	0.3 mm	88%
	Kidney (right)	89%	0.1 mm	0.5 mm	0.3 mm	88%
	Bladder	89%	0.1 mm	0.4 mm	0.2 mm	89%
	Intestine	88%	0.3 mm	1.3 mm	0.6 mm	87%
**Contrast-enanced micro-CT**	Spleen	89%	0.1 mm	0.5 mm	0.2 mm	88%
	Heart	92%	0.1 mm	0.5 mm	0.2 mm	91%
	Lung	95%	0.0 mm	0.2 mm	0.1 mm	94%
	Liver	91%	0.2 mm	0.9 mm	0.3 mm	91%
	Kidney (left)	90%	0.1 mm	0.6 mm	0.3 mm	88%
	Kidney (right)	86%	0.2 mm	0.8 mm	0.4 mm	85%
	Bladder	88%	0.1 mm	0.5 mm	0.2 mm	87%
	Intestine	87%	0.3 mm	1.8 mm	0.9 mm	85%
	Stomach	79%	0.3 mm	1.4 mm	0.8 mm	77%
	Bone	92%	0.0 mm	0.1 mm	0.4 mm	91%
**Light-sheet fluorescence microscopy**	Brain	88%	0.1 mm	1.2 mm	0.4 mm	86%
	Heart	89%	0.1 mm	0.6 mm	0.2 mm	88%
	Lung	84%	0.1 mm	0.7 mm	0.3 mm	82%
	Liver	83%	0.1 mm	1.5 mm	0.4 mm	81%
	Kidney (left)	90%	0.1 mm	0.3 mm	0.2 mm	88%
	Kidney (right)	92%	0.1 mm	0.4 mm	0.2 mm	91%
	Spleen	73%	0.1 mm	1.4 mm	0.4 mm	69%

Supplementary Figure S4. Detailed performance scores. Extending the data provided in Figures 2 and 6, this table lists detailed performance scores of AIMOS for all organs and datasets (Dice score, 50th and 95th percentile of the Hausdorff distance, center of mass displacement). Furthermore, the right column provides the Dice scores of AIMOS if the optional ensemble-voting is de-activated.

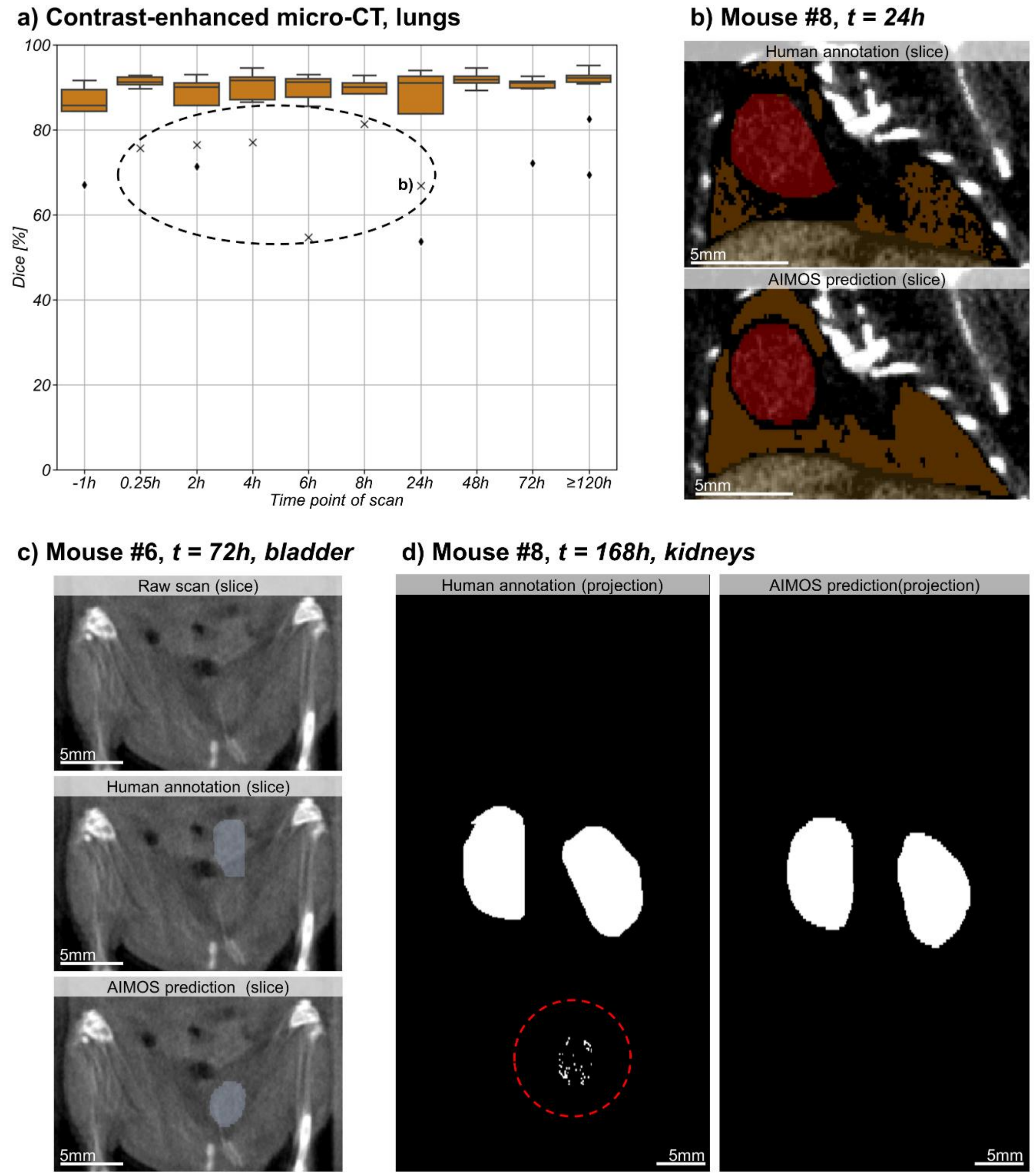

Supplementary Figure S5. Human labels may be inaccurate. Even when AIMOS predicts accurate segmentations, the performance scores may be low due to inaccurate references. **a)** Box plot of prediction performance (Dice score) per time point for the lungs in contrast-enhanced CT dataset. The number of independent scans and the number of biologically independent animals varies by time point and is provided in Supplementary Figure S2b). Each box extends from lower to upper quartile values of data, with a black line at the median; whiskers extend to outmost data point within 1.5x the interquartile range; outliers are represented by crosses for mouse #8 and as diamonds for all other mice. Outliers mostly occur in mouse #8. **b)** Example for mouse #8 for time point t = 24h. Despite a good predicted segmentation of the lungs, the reported Dice score (65%) is low due to inaccurate human annotations. **c)** In one case (mouse #6, t = 72h), a complete mismatch between the AIMOS prediction for the bladder and the human reference revealed that the human expert had misplaced the label. **d)** Erroneously labeled, isolated pixels in the human reference annotation (here shown in red circle for the kidneys of mouse #8, t = 168h) may drastically affect the Hausdorff distance, which measures the distance between segmentation contours. The 50th percentile Hausdorff distance is less sensitive to such cases than the 95th percentile. Source data are provided as a Source Data file.
