# Supplementary material for: Deep learning-enabled multi-organ segmentation in whole-body mouse scans
Source: Nat Commun. 2020 Nov 6;11:5626. doi: 10.1038/s41467-020-19449-7 (PMC7648799; doi:10.1038/s41467-020-19449-7)
Supplement: Supplementary file 2 — Reporting Summary [file 41467_2020_19449_MOESM2_ESM.pdf]

nature research

Corresponding author(s): Oliver Schoppe, Ali Ertürk, Bjoern H. Menze

Last updated by author(s): 28.09.2020

Reporting Summary

Nature Research wishes to improve the reproducibility of the work that we publish. This form provides structure for consistency and transparency in reporting. For further information on Nature Research policies, see Authors & Referees and the Editorial Policy Checklist.

Statistics

For all statistical analyses, confirm that the following items are present in the figure legend, table legend, main text, or Methods section.

n/a	Confirmed	
☐	☒	The exact sample size (n) for each experimental group/condition, given as a discrete number and unit of measurement
☐	☒	A statement on whether measurements were taken from distinct samples or whether the same sample was measured repeatedly
☐	☒	The statistical test(s) used AND whether they are one- or two-sided<br>*Only common tests should be described solely by name; describe more complex techniques in the Methods section.*
☒	☐	A description of all covariates tested
☒	☐	A description of any assumptions or corrections, such as tests of normality and adjustment for multiple comparisons
☐	☒	A full description of the statistical parameters including central tendency (e.g. means) or other basic estimates (e.g. regression coefficient) AND variation (e.g. standard deviation) or associated estimates of uncertainty (e.g. confidence intervals)
☒	☐	For null hypothesis testing, the test statistic (e.g. F, t, r) with confidence intervals, effect sizes, degrees of freedom and P value noted<br>*Give P values as exact values whenever suitable.*
☒	☐	For Bayesian analysis, information on the choice of priors and Markov chain Monte Carlo settings
☒	☐	For hierarchical and complex designs, identification of the appropriate level for tests and full reporting of outcomes
☒	☐	Estimates of effect sizes (e.g. Cohen's d, Pearson's r), indicating how they were calculated

Our web collection on statistics for biologists contains articles on many of the points above.

Software and code

Policy information about availability of computer code

Data collection	Two datasets were used; one is publicly available (Rosenhain et al., 2018) as described in the manuscript; the other was collected as part of a prior study and used software is described in detail in Pan et al., 2019; no further software was used for data collection
Data analysis	Data analysis is central to the research presented; all software used is open source as described in the manuscript; all code developed for data analysis in this study is made freely available on GitHub (https://doi.org/10.5281/zenodo.4048770 [54]) and CodeOcean (https://www.doi.org/10.24433/CO.2308253.v1) as described in the manuscript. The entire pipeline was implemented in Python (v3.6) and only required a small num-ber of open-source packages: PyTorch (v1.0) [43] (deep learning framework), SciPy (v1.3) [57,59,18] (scientific computing), and NiBabel (v2.5) [6] (input/output for volumetric datain the NIfTI file format).

For manuscripts utilizing custom algorithms or software that are central to the research but not yet described in published literature, software must be made available to editors/reviewers. We strongly encourage code deposition in a community repository (e.g. GitHub). See the Nature Research guidelines for submitting code & software for further information.

Data

Policy information about availability of data

All manuscripts must include a data availability statement. This statement should provide the following information, where applicable:

- Accession codes, unique identifiers, or web links for publicly available datasets
- A list of figures that have associated raw data
- A description of any restrictions on data availability

All the imaging data and corresponding annotations used in this study areopen source and freely available online. Both the micro-CT datasets (nativeand contrast-enhanced) including annotations are available at Nature ScientificData [48]. We deposited the light-sheet microscopy dataset (native and withnucleus-staining fluorescent signal from PI) including annotations and all pre-trained models as public datasets on the Harvard Dataverse (https://doi.org/10.7910/DVN/LL3C1R [52];https://doi.org/10.7910/DVN/G6VLZN [53]. Source data are provided with this paper. All further relevant data are availablefrom the authors.

Field-specific reporting

Please select the one below that is the best fit for your research. If you are not sure, read the appropriate sections before making your selection.

☒ Life sciences ☐ Behavioural & social sciences ☐ Ecological, evolutionary & environmental sciences

For a reference copy of the document with all sections, see nature.com/documents/nr-reporting-summary-flat.pdf

Life sciences study design

All studies must disclose on these points even when the disclosure is negative.

Sample size	No sample size calculation was made; we used the maximum number of available samples per data set (native CT: n=140, contrast-enhanced CT: n=81; light-sheet microscopy: n=15). The sample size was deemed sufficient based on the analysis shown in Supplementary Figure S1c), which shows that AIMOS can be effectively trained with the number of samples provided.
Data exclusions	For the contrast-enhanced CT dataset, a total of 4 of 85 samples were excluded as described in the manuscript due to incomplete data (missing annotations and missing time-series)
Replication	The model training was replicated over n>10 times. Every attempt was successful. All data, code, and the trained models are publicly available to allow for full replicability. To further foster adoption and lower barriers to reproduce our work, a fully functional online version of the processing pipeline is provided on CodeOcean.
Randomization	Scans from mice were randomly assigned to training, validation, and testing splits of the dataset in a k-fold-cross-validation procedure to assess generalization performance of the processing pipeline; this follows established procedures in the field.
Blinding	Blinding is not relevant in this study as there are no experiment and control groups; the k-fold-cross-validation procedure ensures unbiased and complete assessment of the entire dataset

Reporting for specific materials, systems and methods

We require information from authors about some types of materials, experimental systems and methods used in many studies. Here, indicate whether each material, system or method listed is relevant to your study. If you are not sure if a list item applies to your research, read the appropriate section before selecting a response.

Materials & experimental systems

n/a	Involved in the study	
☒	☐	Antibodies
☒	☐	Eukaryotic cell lines
☒	☐	Palaeontology
☐	☒	Animals and other organisms
☒	☐	Human research participants
☒	☐	Clinical data

Methods

n/a	Involved in the study	
☒	☐	ChIP-seq
☒	☐	Flow cytometry
☒	☐	MRI-based neuroimaging

Animals and other organisms

Policy information about studies involving animals; ARRIVE guidelines recommended for reporting animal research

Laboratory animals	Species: mice; strains: NSG (NOD/SCID/IL2 receptor gamma chain knockout), NMRI nu/nu mice, C57BL/6; sex: male and female; age: 1-4 months. Animals were housed in our animal facility under a 12/12 h light/dark cycle and were provided food and water ad libitum. The mice were housed in our animal facility under a 12/12 h light/dark cycle with food and water ad libitum. The ambient temperature was kept at 20-22°C with 45-55% humidity.
Wild animals	No wild animals were used.
Field-collected samples	No field-collected samples were used.
Ethics oversight	Institutional Animal Care and Use Committees (IACUC) of Technische Universität München, Ethical Review Board of Regierung von Oberbayern; UK Home Office; veterinary department of the regional council in Darmstadt, Hesse, Germany;

Note that full information on the approval of the study protocol must also be provided in the manuscript.
